# A patient-specific three-dimensional couplant pad for ultrasound image-guided radiation therapy: a feasibility study

**DOI:** 10.1186/s13014-018-1098-7

**Published:** 2018-09-03

**Authors:** Heejung Kim, Ah Ram Chang, Sungwoo Cho, Sung-Joon Ye

**Affiliations:** 1 Department of Biomedical Engineering, Seoul National University, Seoul, Korea; 2 Department of Radiation Oncology, Soonchunhyang University Hospital, Seoul, Korea; 3 Department of Surgery, Soonchunhyang University Hospital, Seoul, Korea; 4 Department of Transdisciplinary Studies, Seoul National University, Seoul, Korea; 5 Advanced Institutes of Convergence Technology, Seoul National University, Suwon, Korea; 6 Biomedical Research Institute, Seoul National University Hospital, Seoul, Korea

**Keywords:** 3D couplant, Ultrasound, IGRT, 3D printing

## Abstract

**Background:**

A wide application of ultrasound for radiation therapy has been hindered by a few issues such as skin and target deformations due to probe pressure, optical tracking disabilities caused by irregular surfaces and inter-user variations. The purpose of this study was to overcome these barriers by using a patient-specific three-dimensional (3D) couplant pad (CP).

**Methods:**

A patient skin mold was designed using a skin contour of simulation CT images and fabricated by a 3D printer. A CP was then casted by pouring gelatin solution into a container accommodating the mold. To validate the use of the CP in positioning accuracy and imaging quality, phantom tests were carried out in our ultrasound-based localization system and then daily ultrasound images of four patients were acquired with and without the CP before treatment.

**Results:**

In the phantom study, the use of CP increased a contrast-to-noise ratio from 2.4 to 4.0. The positioning accuracies in the US scans with and without the CP were less than 1 mm in all directions. In the patient study, the use of CP decreased the centroid offset of the target volume after target position alignment from 4.4 mm to 2.9 mm. One patient with a small volume of target showed a substantial increase in the inter-fractional target contour agreement (from 0.07 (poor agreement) to 0.31 (fair agreement) in Kappa values) by using the CP.

**Conclusions:**

Our patient-specific 3D CP based on a 3D mold printing technique not only maintained the tracking accuracy but also reduced the inter-user variation, as well as that could potentially improve detectability of optical markers and target visibility for ultrasound image-guided radiotherapy.

## Background

Ultrasound (US) can provide a superior soft tissue contrast and a high-spatial resolution (i.e. in the sub-millimeter range) [1]. In addition, US needs no extra radiation exposure for imaging, which potentially allows a daily image guidance over the course of radiation therapy (RT). One of the most widely used US applications in RT is a B-mode acquisition and tracking system (BAT; Nomos, Cranberry Township, PA, USA), which was first introduced in the 1990s. The most up-to-date US imaging system for IGRT is Clarity® (Elekta Ltd., Montréal, Québec, Canada). The advantage of this system over the conventional BAT system is that it eliminates inter-modality discrepancy by installing US devices in both a CT simulation room and a treatment room.

However, there have been a few issues in ultrasound image-guided radiation therapy (US IGRT). First, its main difference with the diagnostic US is the optical position-tracking system. Since the optical tracking system fixed on the ceiling should detect the reflective marker attached on the probe in the same coordinates as in the treatment room, the tracking range of the optical tracking system is limited by such geometry [2, 3]. In addition, the probe rotation is limited due to the marker position. Since the markers should be facing toward the camera, irregularities and steep slopes of patient’s skin hinder detectability of optical markers. Second, to improve the image quality of US, the air gap between the probe and the patient’s skin must be removed. For this, during the US scan the pressure should be applied to the probe, which may deform the scanning region and distort images. As a result, target positional discrepancies in US images between the simulation and the treatment might be a concern [1, 4–7]. In order to minimize the probe pressure when the target lies underneath the skin or under an irregular surface, copious US gel and careful probe movement must be applied [8]. Third, the pressure manually applied to the US probe significantly varies depending on the users and thus influences the image quality of US. Such inter-personal variation has been an issue in US IGRT systems [7]. Moreover, US imaging involves the manual movement of a probe device over potentially irregular patient surfaces, which can be an another source of inter-personal variation. Therefore, achieving consistent and reproducible US scanning requires adequate user experience and training [4, 5]. Since an RT course consists of multiple fractions scheduled over 7–8 weeks, it is difficult even for a skilled user to maintain scanning consistency over the entire treatment period [9]. Forth, B-mode US is inherently limited by the dead zone artifact that occurs around the imaging region near the probe, depending on the probe frequency [10]. In order to avoid the acoustic noise near the transducer and to bring the lesion into the focal zone (geometric focus) of probe and out of the dead zone, a sufficient thickness of US coupling gel above the patient’s skin has to be used especially for the superficial targets [11].

Conventionally, US gel, nipple pads and gel pads have been used as a coupling material. However, for irregular surfaces, the viscosity of US gel is too low. Nipple pads can be applied, but are limited to the breast nipple. Alternatively, gel pads (pad-type acoustic coupling materials) are commercially available with high prices. To facilitate US scanning on irregular surfaces, coupling pads of various materials have been investigated [12–17]. However, due to their very low viscosity, these pads require additional gels in some cases to remove air gap between the probe and patient skin surface. With their standard size, fixed thickness and shape, they are not flexible enough to fit over the scanning area of skin surface for each patient. To overcome the aforementioned problems in US IGRT, this study developed a patient-specific 3D couplant pad (CP) with an aid of 3D printing technology. We optimized the design and composition of the pad to facilitate its use in US IGRT. Finally, we investigated the feasibility of our CP and its clinical application through phantom and patient studies.

## Methods

### Equipment for IGRT

In this study, the US system used for IGRT was Clarity® 3.1 (Elekta Ltd., Montréal, Québec, Canada). Its main difference from the diagnostic US is the optical position-tracking system that consists of a ceiling-mounted optical camera system and eight infrared reflecting markers attached to the probe. This optical tracking system with the probe was calibrated with respect to the room reference coordinate by using the specific US calibration phantom (provided by the manufacturer). During the US scanning, it detects the marker position to determine the target location. The CT simulation scanner and the LINAC system used in this study were Philips Brilliance Big Bore 16-slice CT-simulator (Philips Healthcare, Cleveland, OH, USA) and Elekta Infinity™ (Elekta AB, Stockholm, Sweden), respectively.

### Couplant pad (CP)

A couplant material should have appropriate acoustic properties to avoid image artifacts. An acoustic property (e.g., speed of sound) can be computed by measuring a signal trigger time to signal response time with the distance to a specific target. However, Clarity US probe acts as both a transmitter and receiver. It does not only have a functionality in clinical mode to measure the signal trigger and response time, but also it does not support any kind of hydrophone connection to be synchronized to Clarity transducer. According to the literatures [18, 19], the water-based gel has the desired ultrasonic transmission properties. Gelatin has been known to have a speed of sound from 1500 m/s to 1600 m/s and its density of 1 g/cm^3^ with low attenuation (0.05 dB/cm*MHz). Thus, in this study, gelatin was selected for the CP material.

The CP making process was as follows: (1) the MIM® (MIM Software Inc., Cleveland, OH, USA) software was used to contour the skin and the treatment target on the CT images and to export the contour as a dicom file; (2) the dicom file of the patient skin contour was imported into the 3D Slicer (BWH and 3D Slicer contributors) software, which was used to make a skin surface model using the SlicerRT module and to convert it to the STL file format for 3D printing [20, 21]; (3) by using a 3D printer (CubePro®, Cubify, 3DSYSTEMS, Rock Hill, SC, USA), the patient skin mold for the area to be scanned by US was made using PLA (Polylactic acid); (4) the edible gelatin powder was mixed with hot water and 83% ethanol as a preservative with the ratio of 1:5:0.5; (5) the patient skin mold was fixed in the container; (6) the gelatin solution was poured into the container and put into the refrigerator for 2 h for solidifying; (7) finally the CP was separated from the container and the patient skin mold. The minimum depth of the CP should be 1 cm or more in order to bring the target into the focal zone. According to the literature, oil can be used as a low viscosity coupling agent substitute for ultrasound gel [22]. To achieve a better contact between the phantom and the CP, baby oil was used. After spreading oil (mineral or vegetable) inside the CP or on the patient skin, the CP was put on the patient. At first US scan, skin markings were done at the CP edges. The CP was positioned by the skin marks and then the US scan was performed. All US scans for all patients were acquired by all three users.

### Phantom study

**Fig. 1:**
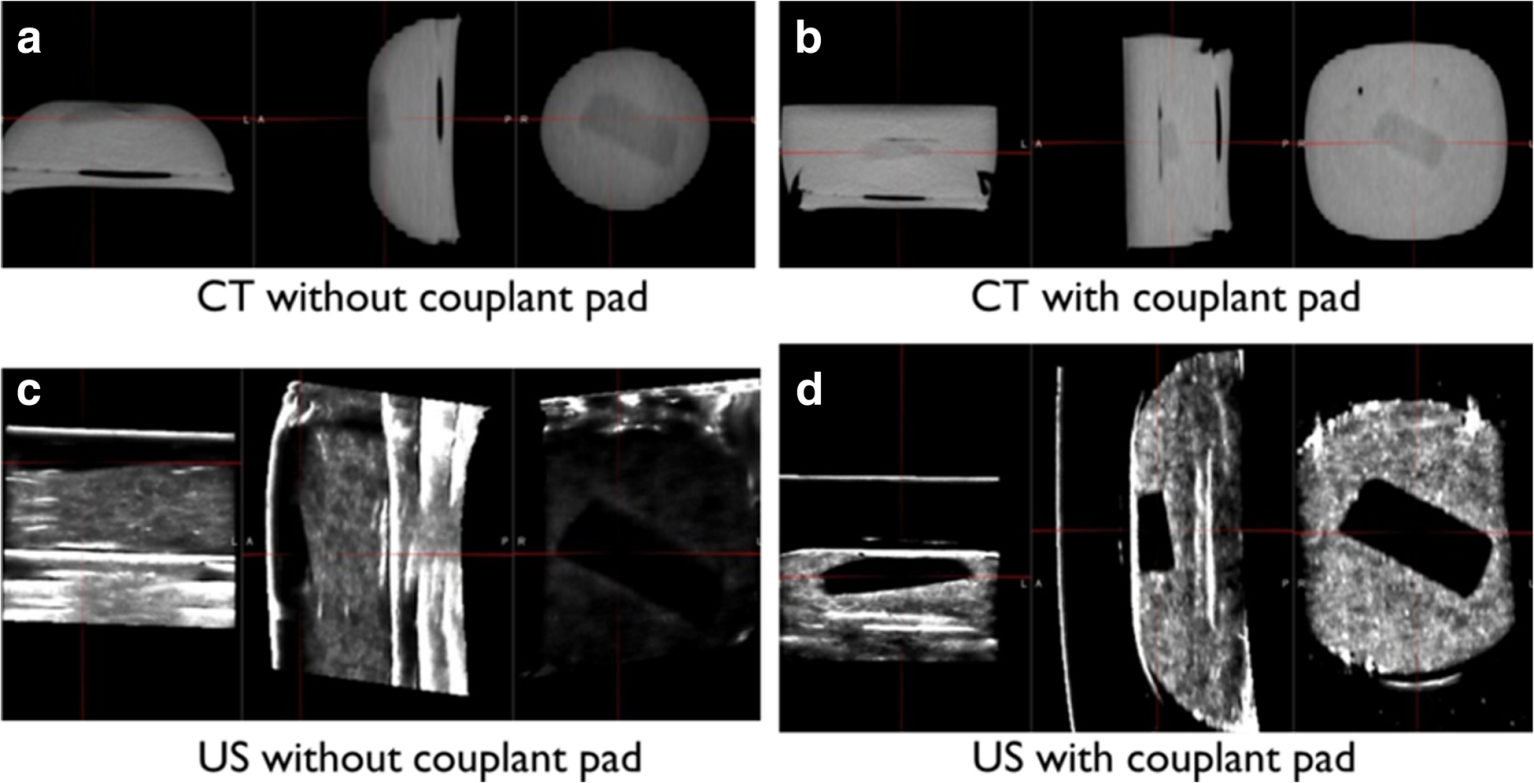
In-house phantom images: **a** The simulation CT image acquired without couplant pad (CP). **b** The simulation CT acquired with CP. **c** The US image acquired without CP. **d** The US image acquired with CP

Prior to the clinical implementation of CP, an in-house phantom was fabricated with a mixture of gelatin and agarose and a CP was fabricated with gelatin only for the phantom study. The mixture of gelatin and agarose was used as a soft tissue and a piece of a commercial gel pad (Aquaflex, Parker Laboratories, INC., Fairfield, NJ, USA) was inserted into the phantom as a target. Figure 1 shows the in-house phantom images acquired using CT and US with and without the CP. US scanning was performed in four different sweeping directions on the CP. Target volume and image contrast were evaluated by contouring volumes of interest (one target and four spheres with 1 cm diameter 2 cm away from target) on CT and US images.

To validate the positioning accuracy of the US-based localization with and without the CP, the Clarity Calibration Phantom (Elekta Ltd., Montréal, Québec, Canada) was used. It was scanned using the simulation CT to be used as a reference. Applying 20 different combination of intentional table shifts (ranged from 1 mm to 30 mm) from the reference location (0, 0, 0) in the left-right (LR), the anterior-posterior (AP) and the inferior-superior (IS) directions, CBCT and US images with and without the CP were acquired.

### Patient study

Among the subjects eligible for US IGRT, four patients were selected in this study to evaluate the effectiveness of the developed CP. The four patients participated in the study after the institutional review board (IRB) approval (IRB number 2014–11-029). Patient 1 had a metastatic inguinal lymph node under an irregular surface. Patient 2 also had a metastatic inguinal lymph node at the depth of about 2 cm from the skin. Patient 3 had a tonsil cancer. Due to the surface curvature of this patient’s head and neck, the probe movement had a difficulty; the probe marker was not detectable by the shoulder. Patient 4 had a soft tissue sarcoma on the shoulder placed at the shallow depth underneath the irregular surface and thus it was difficult to apply pressure with the probe. The curved probe was used for patients 1 and 3 and the linear probe was used for patients 2 and 4.

For all patients, the US scanning was performed at every treatment day before the treatment and the x-ray image acquisition was performed minimum once a week and maximum twice a week before the treatment by using the CBCT. For four patients (patient 1–4), the simulation CT image, US images with and without the CP and CBCT images were acquired. Due to the patient’s inevitable condition such as a deterioration of general condition and a low CBC (complete blood count) level that is not available to treat and due to the patient’s treatment refusal, total 324 US images and 16 CBCT images were acquired in 21, 18, 6 and 9 fractions for patient 1, 2, 3 and 4. The US scanning time except of image registration process and patient setup was evaluated.

### Data analysis and statistics

US images of four patients (patient 1–4) were acquired before the treatment by three different users (one physicist, two therapists) in both conditions with and without applying the CP. The first US image (taken at the first treatment day) for each patient was used as a reference and all US images were registered to it. The target volume (GTV) was contoured on the US images. The volume of the target and the target centroid shift were calculated. The contrast-to-noise ratio (CNR) was calculated an area inside and 3 mm outside the target volume in the US images. The variations in the volume of the target due to the deformation of the target depending on the US scanning conditions were also evaluated. Each target volume difference (%) was calculated by dividing a difference between the target volumes in the first US image and each US image by the target volumes in the first US image. Inter-user variation in US scanning was evaluated by analyzing the centroid shift of the target. The CERR (Computational Environment for Radiotherapy Research) software was used in order to evaluate the level of agreement among the target contours in terms of kappa values calculated using the Fleiss’ eq. [23–25]. Using the SPSS software, a linear mixed model was calculated to investigate the developed CP in terms of image contrast and inter-user variation.

## Results

### Phantom study result

The target and surrounding structures (four spheres with 1 cm diameter at 2 cm away from the target) of the in-house phantom were delineated. Compared with the target volume (5.9 ml) in the CT image as a reference, it was not changed (less than 0.1 ml) by the probe sweeping direction in the US image with the CP, while it was varied by up to 1.6 ml in the US image without the CP because the target of the in-house phantom was located superficially. In addition, by using the CP, the CNR on the US image was increased from 2.4 to 4.0, comparing with the CNR (2.8) on the CT image.

Table 1 shows the Clarity Calibration Phantom test result for the localization accuracy of US scanning with and without the CP, comparing with CBCT. The US positioning errors were less than 0.6 mm in all directions, regardless of applying the CP. This result indicates that the Clarity® US image guidance system used in this study had no systematic error for target localization. Compared to CBCT as a ground truth, the US tracking for localization was accurate within 0.6 mm, even when using the CP: 0.1 ± 0.3 mm, 0.1 ± 0.2 mm and − 0.3 ± 0.7 mm for the US without the CP and 0.0 ± 0.2 mm, 0.2 ± 0.3 mm and − 0.6 ± 0.4 mm for the US with the CP in LR, AP and IS direction. For a reference, our couch and CBCT positioning accuracy are maintained to be within 1 mm by our monthly QA based on TG 142 report and the manufacturer engineer’s periodic maintenance.

**Table 1:** Accuracy of US image-based localization with and without couplant pad (CP), comparing with CBCT: positioning errors from phantom test with intentional 3-dimensional table shifts (ranged from 1 mm to 30 mm in each direction)

	LR (mm)	AP (mm)	IS (mm)
CBCT	0.3 ± 0.3	0.5 ± 0.4	0.6 ± 0.5
US without CP	0.4 ± 0.2	0.6 ± 0.4	0.3 ± 0.3
US with CP	0.3 ± 0.2	0.5 ± 0.3	0.2 ± 0.1

*Abbreviations*: *LR* Left-Right, *AP* Anterior-Posterior, *IS* Inferior-Superior, *CBCT* Cone-Beam Computed Tomography, *US* Ultrasound, *CP* Couplant Pad

### Patient study result

**Fig. 2:**
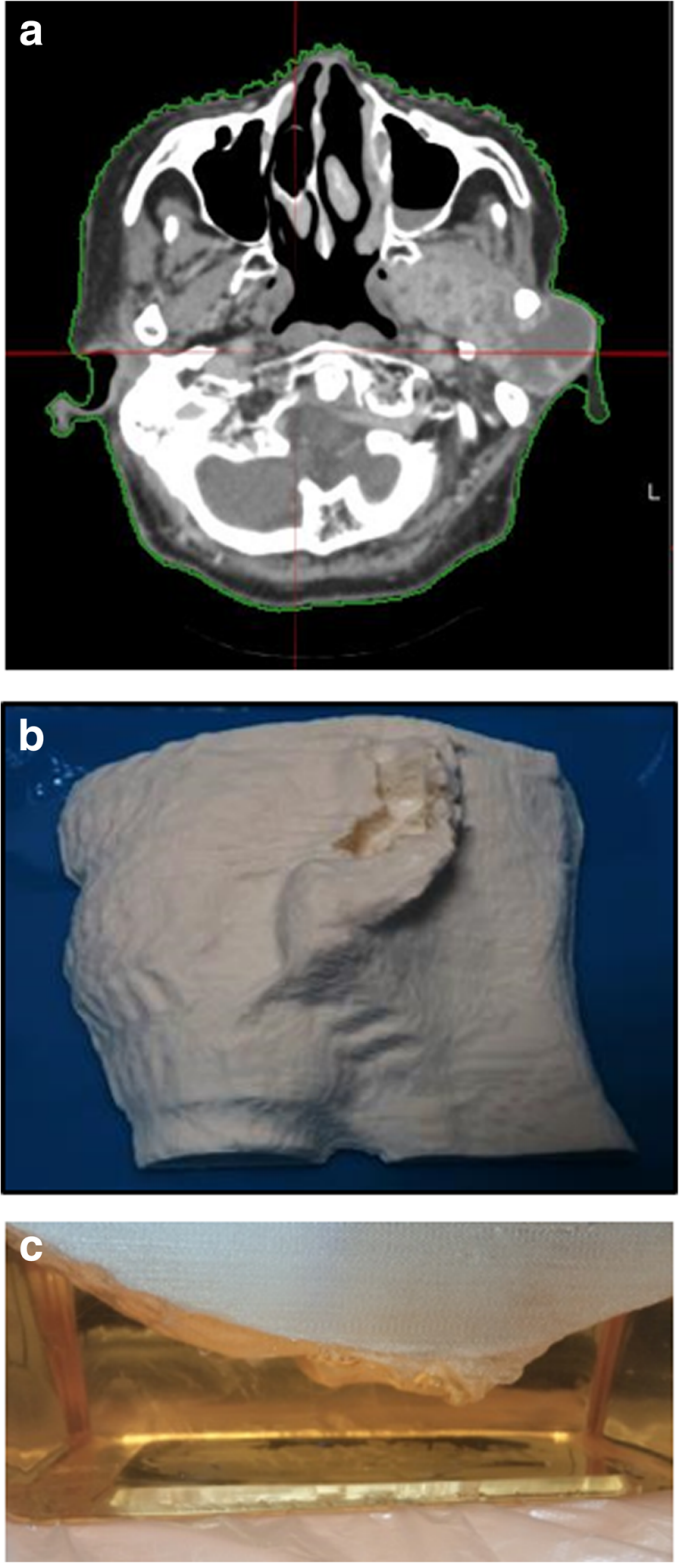
Example of the process for fabricating a patient-specific 3D couplant pad (CP). **a** The patient skin contour is extracted from the simulation CT images. **b** The patient skin mold is fabricated by 3D printer. **c** A CP is casted by pouring gelatin solution into a container accommodating the mold

**Fig. 3:**
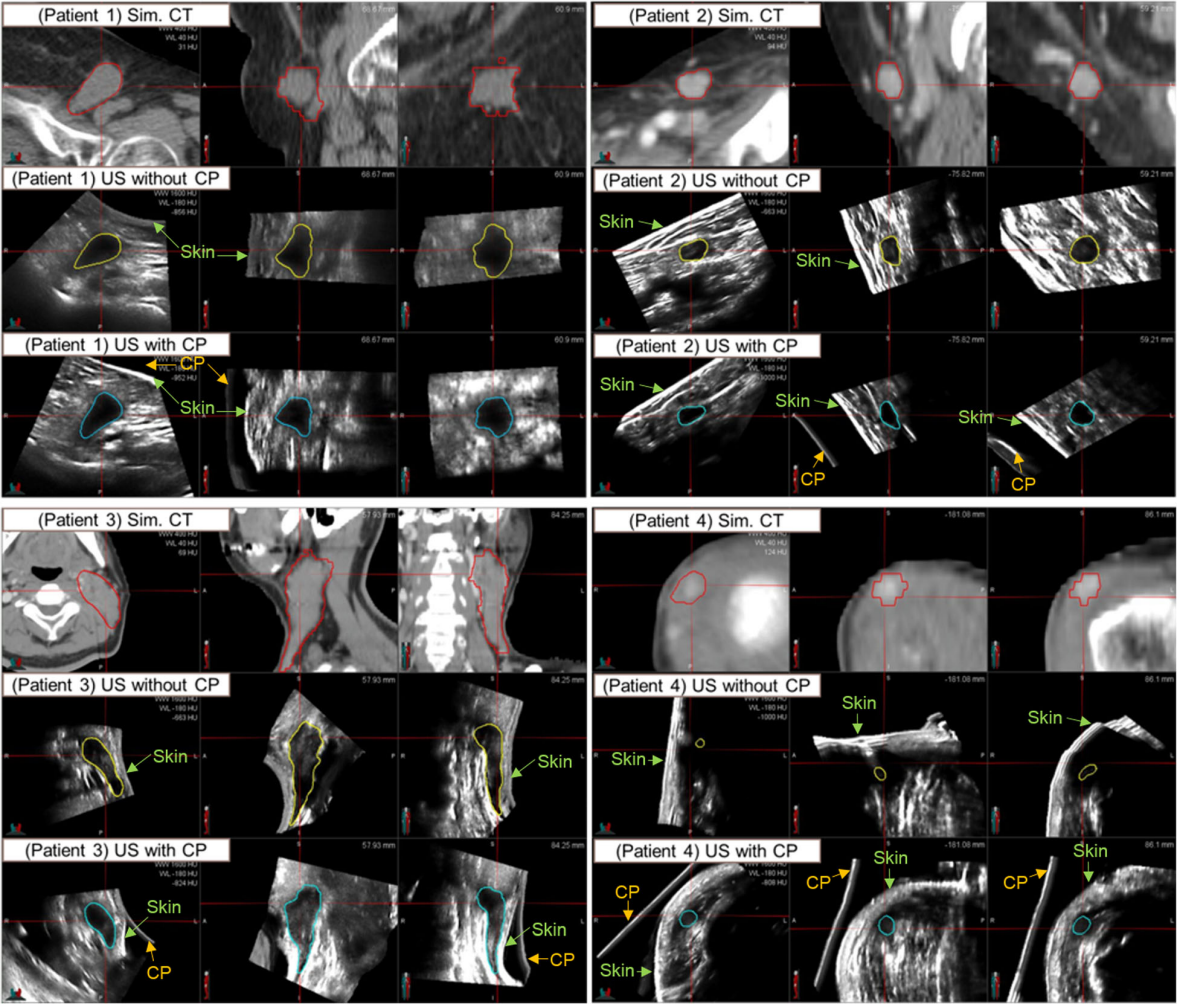
US image acquisitions without and with using CP relative to the simulation CT image for four patients. The targets (GTV) were delineated on each images

As shown in Fig. 2, the patient-specific CP for each patient was fabricated using the 3D printed-patient skin mold. Overall, by the use of the CP, the US hand-held probe could be easily swept on the flat side of the CP. In all patients, when the CP was not applied, a large amount of US coupling gel was needed in order to minimize the probe pressure due to the target being located at a shallow depth from the skin. For patient 4, the irregularity of patient’s skin was excessive, so that the US scan could not cover the whole target region and the dark shadow due to the air was shown around the target. However, when the CP was applied, there was no difficulty for scanning and no need of a large amount of gel. Moreover, the patient’s skin was clearly visible on US images, so that it was easier to fuse the images and to delineate the target. Figure 3 shows the difference between the US scans without and with the CP for all patients relative to their simulation CT images. Individually, for patient 1, the skin deformation due to the pressure from the hand-held probe was removed. For patient 2, the image contrast was improved and the edges of the target were clearly visible. For patient 3, the area between neck and shoulder with high curvature was filled by the CP so that the US scanning area between probe and CP became flat. Thus, the long target volume was scanned completely within the US optical tracking range. For patient 4, the shoulder surface was scanned without any artifact and deformation. In addition, the small superficial target near the shoulder became observable.

Except of image registration process and patient setup, the average US scanning time was 1 min 19 s for US without the CP and 1 min 24 s. For patient 1 and 2, it was less than 1 min 10 s on average regardless of the use of the CP. For patient 3 (large scanning area and large CP), US scanning without the CP took 56 s, but it increased to 1 min 39 s due to setting up the CP. However, for patient 4 (severely curved surface but small CP), it took 3 min 10 s without the CP and decreased to 2 min 20 s due to the use of CP.

**Fig. 4:**
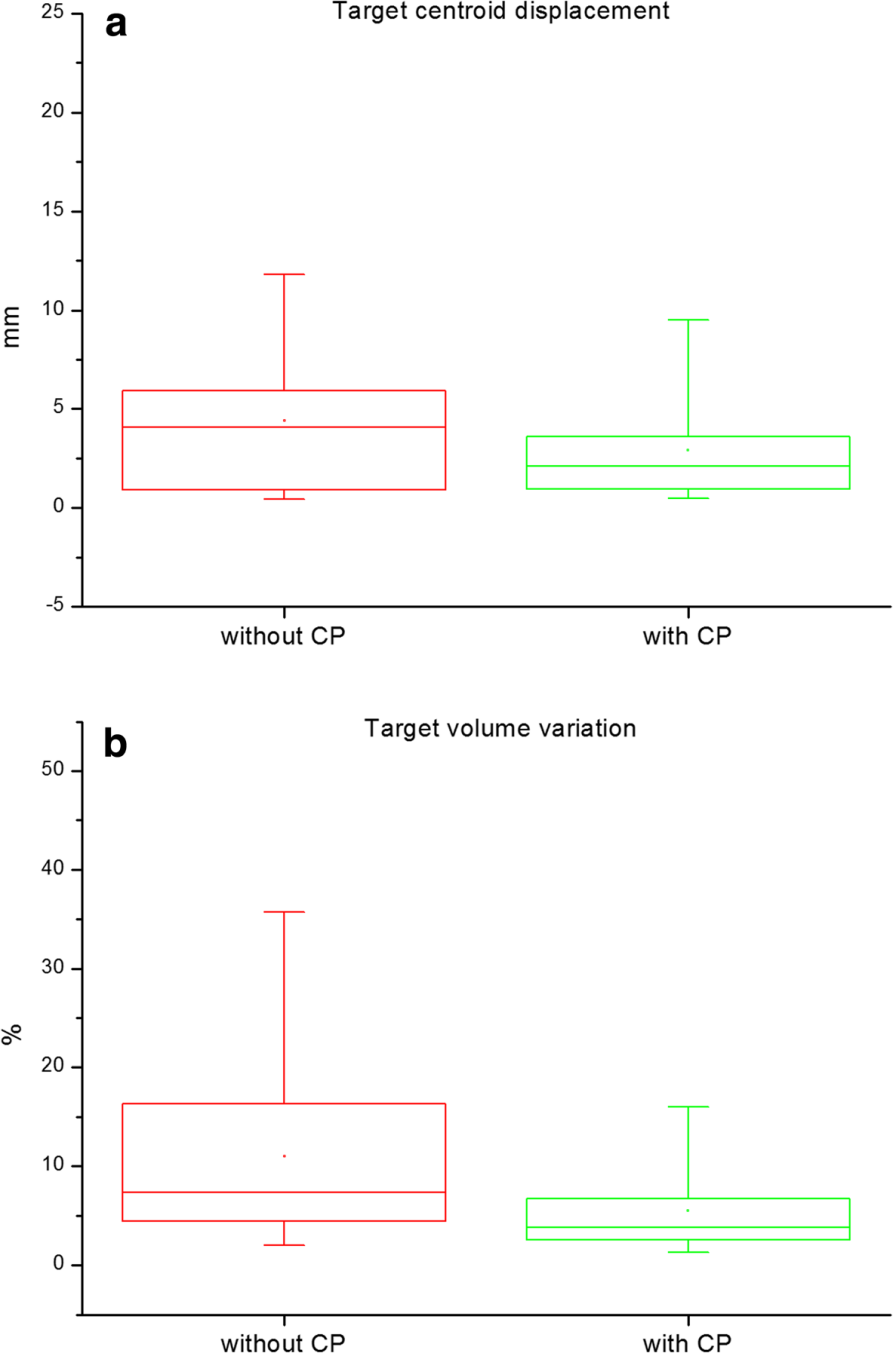
Box-and-whisker charts for the results of (**a**) target centroid displacement and (**b**) target volume variation. The box is determined by the 25th and 75th percentiles and the whiskers are determined by the 5th and 95th percentiles. In the box, the line and small box represent the median and mean values

The centroid shift of target in US images without the CP was − 0.7 ± 2.1 mm in LR and − 0.4 ± 4.2 mm in IS, but 2.1 ± 2.8 mm in AP. However, in US images with the CP it was less than 1 mm in all directions; 0.1 ± 1.7 mm in LR, 0.7 ± 1.7 mm in AP, − 0.6 ± 3.3 mm in IS. As shown in Fig. 4 (a), the 3D vector magnitude of the centroid shift decreased from 4.4 ± 3.9 mm (4.1 mm Median) to 2.9 ± 3.0 mm (2.1 mm Median). This effect of using CP on the centroid shift of target was statistically significant in LR, AP and 3D vector magnitude with *p*-values less than 0.001 for the linear mixed model. As listed in Table 2, the difference among the three different users in the mean and 1 SD values of the centroid offset of target was decreased due to the use of CP. Depending on the user’s skills (e.g., probe pressure and scanning area coverage), the centroid offset was significantly different when the US scan was performed without the CP (*p* < 0.05 in all directions).

**Table 2:** Centroid offset of target after target position alignment: Analysis of the correlation of the centroid offset among the three different users by using the linear mixed model

	User 1	User 2	User 3	*p*-value
Without CP				
LR (mm)	− 0.7 ± 2.2	−0.2 ± 2.1	− 1.2 ± 2.0	0.005
AP (mm)	2.1 ± 2.8	1.5 ± 2.3	2.6 ± 3.2	0.002
IS (mm)	0.2 ± 4.7	0.5 ± 3.9	− 1.8 ± 3.5	< 0.001
3D (mm)	4.5 ± 4.4	3.9 ± 3.5	4.8 ± 3.8	0.020
With CP				
LR (mm)	− 0.0 ± 1.4	0.3 ± 1.7	0.1 ± 1.8	0.081
AP (mm)	0.5 ± 1.4	0.7 ± 1.5	0.8 ± 2.2	0.582
IS (mm)	−1.0 ± 3.2	− 0.6 ± 3.2	− 0.4 ± 3.5	0.194
3D (mm)	2.7 ± 2.9	2.8 ± 2.9	3.3 ± 3.2	0.133

*Abbreviations*: *LR* Left-Right, *AP* Anterior-Posterior, *IS* Inferior-Superior, *3D* the amplitude of three-dimensional vector, *CP* Couplant Pad

The image contrast between the target and the surrounding tissue were compared in terms of the CNR. Only for the patient 2 (small target on shoulder), the CNR was significantly increased from 3.7 ± 1.0 to 4.1 ± 1.1 with *p*-value less than 0.05. Contrary to the phantom study result, overall CNR had no statistically significant increase in the image contrast when applying the CP. It was 3.4 ± 1.5 for US without CP and 3.4 ± 1.3 for US with CP.

The target volume contouring variation among the different users were evaluated. As shown in Fig. 4 (b), the target volume contouring variation was decreased from 11.0% ± 10.2% to 5.5% ± 4.4% due to the use of CP (*p* = 0.001). The agreement of inter-fractional target contours on the registered US images was evaluated in terms of kappa values. For patient 1, 2 and 3, all kappa values were over 0.61 (good agreement) regardless of the use of CP. For the patient 4, the target volume was very small (about 0.2 ml) and the US scanning without the CP was very difficult because the target was placed under shoulder (steep and curved surface). The kappa values in US images without the CP were less than 0.07 (poor agreement); however, the kappa value was increased into over 0.31 (fair agreement) due to the use of CP.

The patient setup errors of US image-based localization with and without the CP were compared with CBCT which is a current gold standard method. As shown in Table 3, the average differences between CBCT and US localizations were less than 2 mm in all directions. In US without the CP, the maximum difference was − 1.9 mm on average in AP direction. When using the CP, it decreased by 1.1 mm. The 3D error magnitude decreased by 0.5 mm.

**Table 3:** Comparison of patient setup errors between CBCT and US image-based localization with and without couplant pad (CP)

	CBCT vs. US without CP	CBCT vs. US with CP
LR (mm)	− 0.3 ± 2.3	0.3 ± 1.9
AP (mm)	−1.9 ± 1.8	−0.8 ± 1.6
IS (mm)	0.8 ± 1.4	1.3 ± 1.2
3D (mm)	3.4 ± 1.4	2.9 ± 1.2

*Abbreviations*: *LR* Left-Right, *AP* Anterior-Posterior, *IS* Inferior-Superior, *3D* the amplitude of three-dimensional vector, *CBCT* Cone-Beam Computed Tomography, *US* Ultrasound, *CP* Couplant Pad

## Discussion

Since the Clarity US system used in this study can use US images as reference images for image registration, no inter-modality effects were present and the scanning direction effects were negligible (less than 0.1 cc). In this study, the CP was made of gelatin, a homogeneous colloid gel that is produced from animal collagen. The collagen was combined with a sufficient amount of water so that the density of the CP ranged from 1004 kg/m^3^–1024 kg/m^3^, which is similar to that of water. Thus, the speed of sound in the CP (1500 m/s – 1600 m/s) may be similar to that in tissue (1520 m/s - 1650 m/s) [19, 26]. The gelatin was easy to use, cost-effective, homogeneous, and exhibited low acoustic attenuation. These highly desirable properties make gelatin an ideal material for a CP to be used during US scanning. To remove the air between the CP and the patient surface, conventional US gel has a higher viscosity than water or oil and did not sufficiently remove the air. The mineral oil yielded a clear interface and did not make air bubbles.

Ultrasound imaging noise is caused by excess gain or low gain [27]. This issue can be resolved by decreasing or increasing the overall gain. However, excessive gain can result in false echoes or oversaturation. To resolve low gain artifacts, applying more acoustic coupling material can help to increase the far gain and the overall gain. For these reasons, CNR for image contrast in the phantom test of this study was increased from 2.4 to 4.0 due to the use of the CP. Unlike the phantom study result, it was not significantly different in the patient study result when applying the CP. Only for the patient 2 who had a small target on shoulder, the CNR was increased from 3.7 to 4.1 (*p* < 0.05). An increase in noise in a target can be a possible reason of this different result for the CNR because the inside of target is very homogeneous in the phantom but heterogeneous in the patients.

The patient-specific 3D CP developed in this study can solve many problems that have been raised regarding the use of the Clarity® US system for IGRT. The specific contour of patient’s skin above the target was used to make the mold for the CP. Theoretically, since the CP has a flat surface on the side where the probe can move with a constant pressure, the marker is always detectable without a dead angle. As in patient 3, the long and highly curved target volume between neck and shoulder was scanned without any difficulty to detect the reflective markers by filling the scanning area with the CP to become a flat surface. With the CP, the gel is used only for the probe surface. As experienced in the cases of patient 3 and patient 4, the flat surface of the CP faced to the US probe can potentially reduce the scanning time and remove the dependence on sweeping directions and multi-path artifact. In this study, the time required to perform a US scan with the CP decreased from 3 min 10 s to 2 min 20 s due to the use of CP for the patient 4. For other patients, it was within 2 min, which is similar to CBCT. As shown in the case of patient 4, the inherent depth of the CP provided an additional benefit of removing dead-zone artifacts. Moreover, the contrast of US images was improved for patient 2 and inter-user variation in daily US scanning was mitigated in all patients. The CP developed in this study was conveniently fabricated with a lower cost (about 20 oz. and $4 per CP) than the commercial gel pad (about 5 oz. and $10 per price), requiring only 3 h from the extraction of patient’s skin contours to the generation of the final CP. When a target volume is changed due to a patient’s weight loss or tumor shrinkage, a planning CT can be re-acquired according to a radiation oncologist’s decision and the CP also can be re-fabricated. In this study, there was no patient who needs the re-planning CT scan during entire treatment procedure.

The US localization accuracy comparing to CBCT has been studied but most studies were about prostate [28–34]. According to the literature which compared the setup errors between CBCT and Clarity transabdominal US, it resulted in the best agreement in LR direction and the largest discrepancy in US toward posterior due to the shift by 2.8 mm in AP direction [32]. According to Wong’s breast US study, the skin displacement due to probe pressure was 3.0 mm compared to CT [35]. The results of US without CP versus CBCT in this study showed the maximum discrepancy (1.9 mm) in AP direction. Although this discrepancy was reduced to 1.3 mm due to the use of the CP, US is experimental up to the present and the patient setup verification using a standard methods (e.g. CBCT) should be performed additionally at least weekly. In addition, as shown in the result of the phantom test in this study, the US localization accuracy was not impaired even with the CP. Unfortunately, no literature exists on the topic of US CPs for US-IGRT. We could not compare our findings to other studies.

This study has some limitations. This findings were based on total 324 images from only four patients and thus additional images from more patients will be required to determine an appropriate PTV margin for our US IGRT. In this study, the 1st treatment US image was used as a reference US image for the image registration. Since the CP was generated from the simulation CT image, the CP was not available for a simulation US scan during a simulation CT scan. If the re-simulation CT and simulation US images using the CP are acquired at the same time before the treatment, the simulation US image can be used as a reference US image for the entire course of treatments. Alternatively, a 3D scanner can be used to make a patient-specific CP before the simulation CT/US.

Unlike the CBCT, the US does not provide an entire body or skin images. In this study, the skin marks for the CP edges were necessary to setup the CP on the patient surface. In addition, it could be difficult to place and fix the CP on the patient surface in a case like a breast which it slides off the skin. The supporting device for the CP can be helpful to immobilize the CP position and it is under development.

There is a limitation of the CP’s lifespan. Once made, it should be put in a separate container and be stored in a refrigerator so that it can be used for up to 2 weeks safely without deformation. As long as the time that the CP is exposed to an air increase, the water consisting of the CP decrease and the CP can shrink. In addition, the ethanol is used as a conservative for fabricating the CP; nevertheless, when the CP is left at the room temperature or it is used more than 3 weeks, it can get moldy. Thus, the gelatin-based CP fabricated by our method in this study should be stored in an airtight container at a low temperature and it is proper to use within 2 weeks. If a sterilized separate space for a CP fabrication, a 3D printer, and a refrigerator for a CP storage are prepared, it will be practical to use it in an RT department for many patients. A new CP material for fabricating once and using it during entire treatment courses will be developed in our further study.

## Conclusions

The patient-specific 3D CP based on the 3D mold printing technique not only maintained the tracking accuracy but also reduced the inter-user variation. This is a promising strategy that could potentially improve detectability of optical markers and target visibility. With taking advantage of non-ionizing radiation, ultrasound technology with this CP could potentially be used for a broader variety of radiotherapy targets.

## Abbreviations

3D: 3-dimensional
AP: Anterior-Posterior
CNR: Contrast-to-noise ratio
CP: Couplant pad
CT: Computed tomography
IS: Inferior-Superior
LR: Left-Right
RT: Radiation therapy
SD: Standard deviation
US IGRT: Ultrasound image-guided radiation therapy
US: Ultrasound
